# Polymer Brushes via Surface-Initiated Electrochemically Mediated ATRP: Role of a Sacrificial Initiator in Polymerization of Acrylates on Silicon Substrates

**DOI:** 10.3390/ma13163559

**Published:** 2020-08-12

**Authors:** Monika Flejszar, Paweł Chmielarz, Karol Wolski, Gabriela Grześ, Szczepan Zapotoczny

**Affiliations:** 1 Department of Physical Chemistry, Faculty of Chemistry, Rzeszow University of Technology, Al. Powstańców Warszawy 6, 35-959 Rzeszów, Poland; d442@stud.prz.edu.pl; 2 Faculty of Chemistry, Jagiellonian University, Gronostajowa 2, 30-387 Kraków, Poland; wolski@chemia.uj.edu.pl (K.W.); gabriela.grzes@doctoral.uj.edu.pl (G.G.); zapotocz@chemia.uj.edu.pl (S.Z.)

**Keywords:** sacrificial initiator-assisted *se*ATRP, silicon wafers, hydroxethyl acrylate, (co)polymer brushes, atomic force microscopy

## Abstract

Silicon wafers as semiconductors are essential components of integrated circuits in electronic devices. For this reason, modification of the silicon surface is an important factor in the manufacturing of new hybrid materials applied in micro- and nanoelectronics. Herein, copolymer brushes of hydrophilic poly(2-hydroxyethyl acrylate) (PHEA) and hydrophobic poly(*tert*-butyl acrylate) (P*t*BA) were grafted from silicon wafers via simplified electrochemically mediated atom transfer radical polymerization (*se*ATRP) according to a surface-initiated approach. The syntheses of PHEA-*b*-P*t*BA copolymers were carried out with diminished catalytic complex concentration (successively 25 and 6 ppm of Cu). In order to optimize the reaction condition, the effect of the addition of a supporting electrolyte was investigated. A controlled increase in PHEA brush thickness was confirmed by atomic force microscopy (AFM). Various other parameters including contact angles and free surface energy (FSE) for the modified silicon wafer were presented. Furthermore, the effect of the presence of a sacrificial initiator in solution on the thickness of the grafted brushes was reported. Successfully fabricated inorganic–organic hybrid nanomaterials show potential application in biomedicine and microelectronics devices, e.g., biosensors.

## 1. Introduction

Surface-grafted polymer brushes are the systems of macromolecules tethered by one end to a surface sufficiently densely for the chains to adopt extended conformations. The possibility of grafting well-defined polymer brushes from inorganic as well as organic surfaces opens the way to modern hybrid systems considered materials of the future. Recent literature reports indicate the versatility of the surface modification methods [1,2,3,4,5]. Particularly interesting are the techniques of reversible-deactivation radical polymerization (RDRP), allowing the control of the density and length of surface-grafted macromolecules at the same time leading to the formation of complex polymer brush architectures, rising from the initiation site attached to the surface according to the *grafting from* approach [6,7,8,9,10]. The *grafting from* method uses the presence of a polymerization initiator connected by a covalent bond to the surface enabling the formation of polymer chains on previously functionalized substrates [11,12,13,14]. 

A wide range of methods have been used for modification of inorganic and organic surfaces with various polymeric brushes, including reversible addition-fragmentation chain transfer polymerization (RAFT) [15,16,17,18,19,20], nitroxide-mediated polymerization (NMP) [21,22,23], as well as atom transfer radical polymerization (ATRP) [4,14,24,25,26,27,28,29,30,31]. The ATRP technique is distinguished by a wide spectrum of advantages making it a useful tool for the synthesis of new functional materials. This technique can be used for the polymerization of a variety of monomers, providing control over the progress of the reaction and resulting in a narrow molecular weight distribution (MWD, *M*_w_/*M*_n_, *Đ*) of the synthesized polymers [32,33,34,35,36,37,38].

In the ATRP, the system strives for an equilibrium through a reversible redox reaction between the form of the catalytic complex where copper is at the first oxidation state (Cu^I^/L) and alkyl halide (P-X) [39,40]. Homolysis of the P-X bond leads to the formation of an alkyl (P*) and halogene radical (X*), which links to the activation complex resulting in its oxidized X-Cu^II^/L form (deactivator) [41,42]. The created radicals can initiate the polymerization process as well as lead to the formation of Cu^I^-species and the “dormant” form of a polymer chain [43,44]. In the case of simplified electrochemically mediated atom transfer radical polymerization (*se*ATRP), the ratio of the catalytically active forms of Cu^I^/Cu^II^ species necessary to prepare homopolymers and copolymer structures is controlled with high accuracy by selecting the applied potential [45,46,47]. The implementation of the *se*ATRP technique enables a significant reduction in the amount of catalytic complex used for controlled polymerization. Therefore, *se*ATRP is considered an environmentally friendly synthetic approach [48,49,50].

There have been no reports presenting sacrificial initiator-assisted controlled grafting of poly(2-hydroxyethyl acrylate) (PHEA) brushes from the surface of silicon wafers. The first literature reports on successful homopolymerization of 2-hydroxyethyl acrylate (HEA) in bulk were presented applying the classical ATRP method (26 957 ppm of Cu) [51]. In 2013, Percec et al. polymerized HEA in situ in a protic solvent (methanol, MeOH; ethanol, EtOH; water, H_2_O) and a dipolar aprotic solvent (dimethyl sulfoxide, DMSO) with the Cu^0^ wire-mediated ATRP technique (957 ppm of Cu) [52]. Further kinetic investigation of both the polymerization of HEA and copolymerization of HEA from a poly(ethylene oxide) initiator in DMSO and H_2_O showed higher monomer conversion for the aqueous process [53,54]. Nevertheless, both tested ligands introduced certain limitations. Application of tris[2-(dimethylamino)ethyl]amine (Me_6_TREN) resulted in an extension of the induction time, whereas *N,N,N’,N’’,N’’*-pentamethyldiethylenetriamine (PMDETA) led to increased dispersity of the PHEA segment [54]. In another example, classical ATRP was used to graft PHEA chains via the *grafting to* approach (5 908 ppm of Cu). Previously polymerized 10 nm end-functionalized PHEA chains were attached to silicon wafers in order to demonstrate the non-biofouling properties of the treated surfaces [55]. Additionally, the *grafting from* method enabled investigation of the controlled nature of polymerization of acrylates via photo-induced copper-mediated radical polymerization (e.g., 473 of Cu) [56]. Moreover, other RDRP methods have been successfully applied to synthesize macromolecular structures containing biocompatible PHEA segments. Self-assembling of poly(2-hydroxyethyl acrylate)-*graft*-poly(methyl methacrylate) copolymers for encapsulation of hydrophilic rhodamine 6G and hydrophobic pyrene were presented using the RAFT and ATRP (857 ppm of Cu) methods [17]. However, a kinetic study of sacrificial initiator-assisted surface-initiated polymerization of HEA has not been performed.

PHEA, thanks to its hydrophilic properties, is an interesting biomaterial with biocompatibility and low cytotoxicity [57,58,59,60]. The developed ultra-low ppm *se*ATRP approach is a new pathway for the synthesis of materials with reduced contamination (several ppm Cu by wt. in an experimental system). Taking into account the existing research and the advantages of the applied method, it is expected that the synthesized copolymers of PHEA-*b*-P*t*BA brushes with hydrophilic and hydrophobic segments will become an important tool for the development of smart surfaces in biomedicine and nanotechnology. Additionally, in the context of microelectronic applications, a P*t*BA block can be easily hydrolyzed to anionic poly(acrylic acid) (PAA) polyelectrolytes.

The main objective of this paper is to present the modification of silicon wafers with PHEA and P*t*BA brushes by means of surface-initiated *se*ATRP in the presence of a sacrificial initiator with a significantly reduced catalytic complex concentration (26 and 6 ppm of Cu).

## 2. Materials and Methods 

### 2.1. Chemicals

Polished silicon wafers were purchased from ON Semiconductor (Rožnov pod Radhoštěm, Czech Republic). 3-Aminopropyl-trimethoxysilane (APTES), 2-bromoisobutyryl bromide (BriBBr, 98%), ethyl 2-bromo-2-methylpropionate (EBIB, 98%), triethylamine (TEA, >99.5%) tetrahydrofuran (THF, >99.9%), acetone (DMK, >99.9%), tetrabutylammonium perchlorate (TBAP, >98%), and sodium bromide (NaBr, >99%) were purchased from Sigma Aldrich (Saint Louis, MO, USA). Dichloromethane (DCM, >99.5%), toluene (>99.5%), methanol (MeOH, >99.8), ethanol (EtOH, 99.9%), hydrogen peroxide (H_2_O_2_, 30%), and sulfuric acid (>95%) were purchased from Chempur (Piekary Śląskie, Poland). *N,N*-Dimethylformamide (DMF, 99.9%) was purchased from Acros (Fair Lawn, NJ, USA). These reagents were used without further purification. Tris(2-pyridylmethyl)amine (TMPA) was synthesized according to a published procedure [61]. A Cu^II^Br_2_/TPMA stock solution was prepared according to reference [62]. 2-Hydroxyethyl acrylate (HEA; 96%; Aldrich) and *tert*-butyl acrylate (*t*BA; >99%; Aldrich) were passed through a column filled with basic alumina prior to use to remove monomethyl ether hydroquinone as an inhibitor. Al wire was purchased from Alfa Aesar (Tewksbury, MA, USA). LDPE cable ties were purchased from LUX-Tools (Wermelskirchen, Germany).

### 2.2. Analysis 

#### 2.2.1. ^1^H NMR Spectroscopy

^1^H NMR spectra in DMSO-*d*_6_ and CDCl_3_ were measured using a Bruker Avance 500 MHz spectrometer. Monomer conversion and theoretical number-average molecular weight (*M*_n,th_) were calculated by NMR [63]. 

#### 2.2.2. Gel Permeation Chromatography (GPC)

Molecular weights (MWs) and molecular weight distributions were measured by GPC (Polymer Standards Services (PSS) columns [guard, 10^5^, 10^3^ and 10^2^ Å]) using the following conditions: THF eluent, 25 °C, flow rate of 1.00 mL/min, and differential refractive index (RI) detector (Viscotek, T60A). The apparent MWs and MWD were determined with a calibration based on PS standards using OmniSEC software from Viscotec Corporation (Malvern, United Kingdom). 

#### 2.2.3. Cyclic Voltammetry (CV)

Cyclic voltammetry was performed using an Autolab model AUT84337 potentiostat running with General Purpose Electrochemical Software (nLab, Warsaw, Poland). The electrolysis was carried out under Ar atmosphere using as a working electrode (WE) a platinum disk (0.071 cm^2^) for CV and platinum plate with a silicon wafer for electrolysis. The platinum disc electrode was carefully polished with 0.05 μm alumina suspension (Buehler) before every single measurement. The reference electrode (RE) was Ag/AgI/I^−^ electrode. The counter electrode (CE) was an Al wire (*l* = 10 cm, *d* = 1 mm) immersed directly in the reaction mixture. CV measurements were conducted in a five-neck electrochemical cell. A vigorous stirring rate (1300 rpm) was applied during the preparative electrolysis.

#### 2.2.4. Atomic Force Microscopy (AFM)

AFM measurements were carried out using a Dimension Icon AFM microscope (Bruker, Santa Barbara, CA, USA) working in the PeakForce Tapping (PFT) and QNM^®^ mode with standard silicon cantilevers for measurements in air (nominal spring constant of 0.4 N/m). In order to determine the thickness of the synthesized polymer layer, AFM images with a total resolution of 256 × 256 pixels were captured at the edge of the scratch made by metal tweezers. The scratched brushes were sonicated in organic solvents before the measurements, and the thicknesses were measured from the obtained depth profiles.

#### 2.2.5. Water Contact Angle Measurements (WCA)

The hydrophilic and hydrophobic characters of the grafted polymer brushes were evaluated by the determination of contact angles values (Θ). Droplets (2 μL) of polar (water, formamide) and nonpolar (diiodomethane) liquids were put on the layer of grafted polymers. The values of the contact angles were determined by the geometric analysis of pictures taken for droplets, which were analyzed by *Kropla* software [64]. For each sample, three droplets of each studied liquid were analyzed. The measurements were carried out at room temperature. Photos of modified silicon surfaces were taken with a Panasonic DC-FZ82 camera equipped with a LUMIX DC VARIO 1:2.8-5.9/3.58-215 ASPH lens. The obtained contact angles were used to determine the free surface energy of the grafted brushes based on the Owens–Wendt method and van Oss–Good method by *Energia* software [65,66].

### 2.3. Surface Functionalization of Silicon Wafers

The ATRP initiator was deposited on silicon wafers in two steps, according to a slightly modified procedure described elsewhere [31]. The silicon substrates were first cleaned by rinsing with ethanol. The dried wafers (under argon) were put into the piranha solution (3:1 mixture of 96% sulfuric acid with 30% hydrogen peroxide) for 1 h. After that, the plates were rinsed with deionized water, THF, and toluene, and dried in a stream of argon. Finally, the substrates were cleaned by a UV–ozone cleaner for 30 min. Such prepared wafers were placed in a flask with 10 mL of toluene. The flask was closed by a rubber septum and then purged with argon. A one drop of APTES was added to the flask with the plates, and the mixture was left to react overnight at room temperature. After that, the substrates were rinsed with a copious amount of toluene, DCM and then additionally sonicated in toluene. In the second step, the plates were immersed in 10 mL of DCM and 0.4 mL of TEA. The flask was closed by a rubber septum and then purged with argon for 10 min. After that, a solution of 2 mL of DCM and 0.37 mL of BriBBr was added dropwise to the flask using argon by double-tipped needles. The reaction was left for 1 h at room temperature under an argon atmosphere. Afterwards, the plates were rinsed with copious amount of DCM and methanol and finally dried in a stream of argon.

### 2.4. Typical Procedure for Synthesis of the PHEA Homopolymer via seATRP under Constant Current Conditions

TBAP (1.09 g, 3.2 mmol), 8 mL of HEA (0.70 mmol), 7.91 mL of DMF (0.10 mol), and 11.2 μL of Cu^II^Br_2_/TPMA stock solution (0.05 M in DMF) were placed into a five-neck electrochemical cell. Except for entry 2, Table S1, 0.1 M NaBr (0.16 g, 1.6 mmol) was additionally introduced. The CV was recorded (Figure S1a–c) using a Pt disk WE, an Ag/AgI/I^−^ RE, and an Al wire CE for determining the appropriate applied potential (Table S1, entry 1: *E*_app_ = *E*_pc_−80 mV vs. Ag/AgI/I^−^; entries 2–3: *E*_app_ = *E*_pc_−120 mV vs. Ag/AgI/I^−^). Then, 76.2 μL of EBiB (0.76 mmol) was injected into the reaction solution and the CV was measured to determine the cathodic response. Subsequently, the Pt plate WE, Al wire CE, and Ag/AgI/I^−^ RE were fitted in the reaction setup and degassed for 30 min. After that, the selected potential was applied using the controlled potential preparative electrolysis method (Figure S2a–c). Samples of the reaction mixture were collected successively to track the monomer conversion using ^1^H NMR. The *M*_n_ and *M*_w_/*M*_n_ were determined by GPC measurements using polystyrene standards and THF as a mobile phase. Before GPC analysis, the sample was acetylated according to a typical procedure for PHEA acetylation.

### 2.5. Synthesis of Si-g-PHEA Brushes via Surface-Initiated SI-seATRP in the Presence of a Sacrificial Initiator

TBAP (4.79 g, 14.0 mmol), 17.5 mL of HEA (152 mmol), 52.3 mL of DMF (0.68 mol), and 167 μL of Cu^II^Br_2_/TPMA stock solution (0.05 M in DMF) were placed into a five-neck electrochemical cell. The CV was recorded (Figure S3) using a Pt disk WE, an Ag/AgI/I^−^ RE, and an Al wire CE for determining the appropriate applied potential (*E*_app_ = *E*_pc_−40 mV vs. Ag/AgI/I^−^). Then, 24.5 μL of EBiB (0.17 mmol) was added to the reaction solution, and the CV was measured to determine the cathodic response. Subsequently, the Pt plate WE, Al wire CE, Ag/AgI/I^−^ RE, and silicon wafers placed in the cable rips inserted in small notches of the rubber septum were fitted in the reaction setup and degassed for 30 min. Afterwards, the selected potential was applied using controlled constant current preparative electrolysis (Figure S4a). After certain time intervals, a cable rip with a silicon wafer was pulled over the surface of the reaction mixture to stop the reaction on the inorganic surface. Aliquots of the reaction mixture were collected successively to track the monomer conversion using ^1^H NMR. *M*_n_ and *M*_w_/*M*_n_ were determined by GPC measurements using polystyrene standards and THF as a mobile phase. Before GPC analysis, the sample was acetylated according to a procedure for PHEA acetylation. Modified silicon wafers were sonicated with DMF and THF, dried under air stream, and then characterized using the AFM technique and contact angle measurements.

### 2.6. Typical Procedure for PHEA Acetylation

The post-reaction mixture was acetylated with 0.5 mL pyridine and 0.1 acetic anhydride for each 20 mg polymer sample. After 16 h of incubation at room temperature, the samples were precipitated with methanol and centrifuged. Dried polymer samples prepared with following procedure were dissolved in THF. The prepared samples were purified by passing through a column filled with neutral alumina and used for GPC measurements. The theoretical *M*_n_ (Table 1, entry 1) was determined based on the molar mass of the acetylated monomer (*M*_n_ = 158.15 × [M]_0_/[I]_0_ × monomer conversion + *M*_EBIB,_ where 158.15 is the molar mass of acetylated 2-hydroxethylacrylate, [M_0_] and [I_0_] describes initial monomer and sacrificial initiator concentration respectively, *M*_EBIB_ is the molar mass of the sacrificial initiator) [52].

### 2.7. Synthesis of Si-g-(PHEA-b-PtBA) Brushes via Chain Extension of Si-g-PHEA Brushes by Ultralow ppm Sacrificial Initiator-Assisted SI-seATRP

TBAP (4.79 g, 14.0 mmol), 28.0 mL of *t*BA (193 mmol), 42 mL of DMF (0.54 mol), and 38.6 μL of Cu^II^Br_2_/TPMA stock solution (0.05 M in DMF) were placed into a five-neck electrochemical cell. The CV was recorded using a Pt disk WE, an Ag/AgI/I^−^ RE, and an Al wire CE for determining the appropriate applied potential (*E*_app_ = *E*_pc_−80 mV vs. Ag/AgI/I^−^). Then, 28.3 μL of EBiB (19.3 mmol) was added to the reaction solution and the CV was measured to determine the cathodic potential. Subsequently, the Pt plate WE, Al wire CE, Ag/AgI/I^−^ RE, and silicon wafers placed in the cable rips inserted in small notches of the rubber septum were fitted in the reaction setup and degassed for 30 min. The selected potential was applied using the controlled potential preparative electrolysis approach (Figure S4d). Aliquots of the reaction mixture were collected successively to track the monomer conversion using ^1^H NMR. *M*_n_ and *M*_w_/*M*_n_ were determined by GPC measurements using polystyrene standards and THF as a mobile phase. Before GPC analysis, the sample was dissolved in THF, passed through a neutral alumina column, dried under vacuum for 12 h, dissolved in methanol, and precipitated by ultrapure water. Coated silicon wafers were washed by sonication with DMF and THF, dried under air stream, and then characterized using the AFM technique and contact angle measurements.

## 3. Results and Discussion

**Scheme 1:**
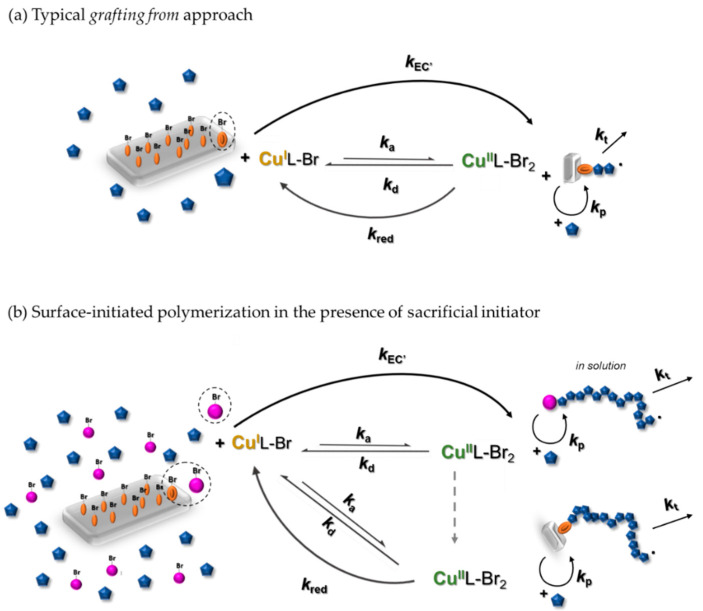
Schematic representation of the ultralow ppm surface-initiated *se*ATRP: without addition of the free initiator in solution (**a**) and sacrificial initiator-assisted process (**b**).

The advantages of the electrochemically mediated polymerization approach include precise control of the Cu^II^/Cu^I^ ratio without the necessity of adding an external reducing agent and the possibility of using an undivided cell (*se*ATRP; sacrificial Al CE) [67]. In this case, the Al wire showed no reduction of the deactivator, because the surface of the Al wire was passivated by forming stable oxidized layers [45]. Some Al was always lost from the CE (2.3–16.6 ppm of oxidized Al for the final HEA/*t*BA polymerization solution; Table S2). However, the theoretical metal concentrations in the pure product were less than 1.8 ppm (Table S2), as a result of the purification procedure in the methanol/water system that was described in earlier works [45,67,68]. In this context, we showed that the sacrificial initiator-assisted SI-*se*ATRP resulting in higher polymerization efficiency compares to SI-*se*ATRP (Scheme 1).

### 3.1. Synthesis of PHEA by Sacrificial Initiator-Assisted SI-seATRP with an Ultralow Content of Copper under Constant Potential Conditions

In order to achieve an appropriate degree of polymerization resulting in a relatively thick brush layer (above 20 nm) the impact of the addition of 0.1 M NaBr on the homopolymerization rate of HEA was investigated (Table S1, entry 2). In essence, the presence of bromide salt slows polymerization (compare *k*_p_^app^; Table S1, entries 1 and 2) due to the shift of dynamic ATRP equilibrium towards the deactivation process [69]. For all reactions, a first-order kinetic plot was observed (Figure S5a–c), and the determined monomer conversion was 27% and 22%, respectively (Table S1, entries 1 and 2), which resulted in DP_app_ less than 300. In order to reach higher monomer conversion and hence thicker PHEA brushes in SI-*se*ATRP and to avoid gelation phenomena, the monomer concentration was decreased. Simultaneously, such procedure resulted in a twofold reduction of the used amount of copper (II) bromide. Nevertheless, a lower concentration of the catalytic complex implied a less controlled process leading to a product with broader MWD (Table S1) [46]. The application of more negative electrochemical potential (*E*_app_ = *E*_p_ −80 mV vs. *E*_app_ = *E*_p_ −120 mV) and decreasing the monomer concentration resulted in a twofold increase in the Cu^I^/Cu^II^ ratio (compare Cu^I^/Cu^II^; Table S3, entries 3 and 5). Furthermore, the dead chain fraction (DCF) value was below 5%, confirming the preserved end-group functionality (Table S4). Finally, further dilution of the monomer (from 50/50 v/v to 25/75 v/v) and *E*_app_ = *E*_pc_−120 mV enabled reaching 31% of the monomer conversion (*k*_p_^app^ = 0.094 h^−1^; Table S1, entry 3). These experimental conditions were applied to prepare PHEA brushes grafted from the silicon wafers.

### 3.2. Synthesis of Block Copolymer Brushes Grafted from the Silicon Surface

For the first time, an ultra-low concentration of copper (II) bromide in the sacrificial initiator-assisted SI-*se*ATRP of hydrophilic and biocompatible HEA and hydrophobic *t*BA was applied (6–26 ppm by wt.; Table 1, entries 1–4). 

**Figure 1:**
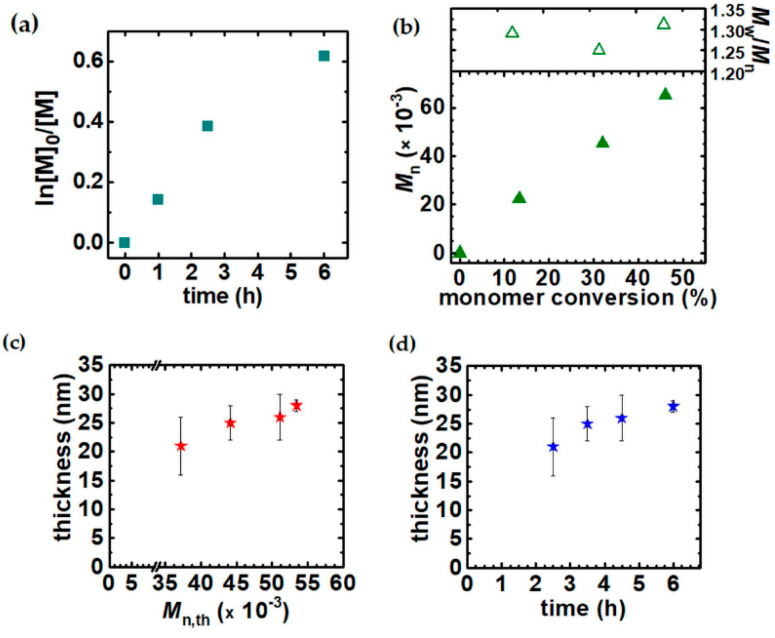
Controlled synthesis of well-defined PHEA brushes grafted from Si-Br. (**a**) First-order kinetic plot of monomer conversion vs. polymerization time. (**b**) *M*_n_ and *M*_w_/*M*_n_ vs. monomer conversion. (**c**) PHEA brush thickness vs. *M*_n,theo_. Table 1, entry 1. (**d**) PHEA brush thickness vs. reaction time. Table 1, entry 1.

A first-order kinetic plot with some deviation in the longest polymerization time was observed for free polymers obtained in the solution during grafting of PHEA brushes from Si-Br (Figure 1a). A linear dependence of *M*_n_ vs. monomer conversion confirmed the controlled nature of the polymerization process (Figure 1b). The application of the appropriate applied potential allowed for control of polymerization and the linear increase in the PHEA brush thickness vs. *M*_n,theo_ of free polymers generated in the solution (Figure 1c) as well as linear dependence of PHEA brush thickness vs. reaction time (Figure 1d). In view of the monomer concertation, the calculated Cu^I^/Cu^II^ ratio was slightly higher but comparable to the result obtained for homopolymerization (compare Table S3, entry 1 and entry 5).

The grafting density (σ) was calculated using the following equation: σ = *N*_A_ × *h* × ρ/*M_n_* [31,71], where *M_n_* is the number-average molecular weight, *N*_A_ is Avogadro’s number, *h* is the measured thickness [25], and ρ = 1.33 g × cm^−3^ is the bulk PHEA density [72]. The calculated value of the grafting density, 0.37 nm^−2^, was determined on the assumption that the polymerization kinetics are similar for the surface-initiated process and polymerization in the solution. Grafting densities of ≈0.25–0.44 nm^−2^ were previously reported for PHEA prepared by photo-induced organotellurium-mediated radical polymerization [73]. The homopolymer brushes demonstrated water contact angles (WCA) of 61.9 ± 1.2°, comparable to literature data for PHEA brushes [74]. The chemical structure of the PHEA chains synthesized in solution (Table 1, entry 1) was confirmed by ^1^H NMR (Figure S8): δ (ppm) = 4.66–4.80 (1H, O*H*–, c), 3.89–4.07 (2H, –C*H*_2_–, a), 3.49–3.63 (2H, –C*H*_2_–, b), 2.14–2.38 (1H, –C*H*=, α), 1.34–1.90 (2H, –C*H*_2_–, β) [75,76].

Herein, assuming that the results obtained for the polymers in solution are equivalent to the brushes, we presented grafting of PHEA brushes from silicon wafers characterized by DCF below 8% (Table S4, entry 1). In order to confirm the preservation of the chain end functionality of grafted PHEA chains, a chain extension test was performed by polymerizing the *t*BA block (Table 1, entry 4 and Figure S9).

The chemical structure of the synthesized P*t*BA chains (Table 1, entry 4) was confirmed by ^1^H NMR (Figure S10): δ (ppm) = 2.14−2.31 (1H, −C*H*=, α), 1.47–1.90 (2H, −C*H*_2_−, β), 1.46–1.38 (9H, C*H*_3_−, b) [26,68]. Chain extension of PHEA brushes was conducted under constant potential conditions (*E*_app_ = *E*_pc_−120 mV). In this case, close to linear first-order kinetic dependence was observed (Figure S9), along with monomodal molecular weight distributions and dispersity equal to 1.81 (Figure S11b). Moreover, the calculated DCF value below 12% confirms preservation of the chain end functionality for PHEA-*b*-P*t*BA and Si-*g*-(PHEA-*b*-P*t*BA) brushes assuming similar polymerization kinetics (Table S4). The water contact angle after grafting of the P*t*BA block increased from 61.9° ± 1.2 to 98.6° ± 1.4 [25,77], confirming the successful polymerization of hydrophobic *t*BA units (Table S5, entry 3 and Figure S12k).

### 3.3. The Impact of the Sacrificial Initiator on the Thickness of the Polyacrylate Brushes Grafted From the Silicon Surface

**Figure 2:**
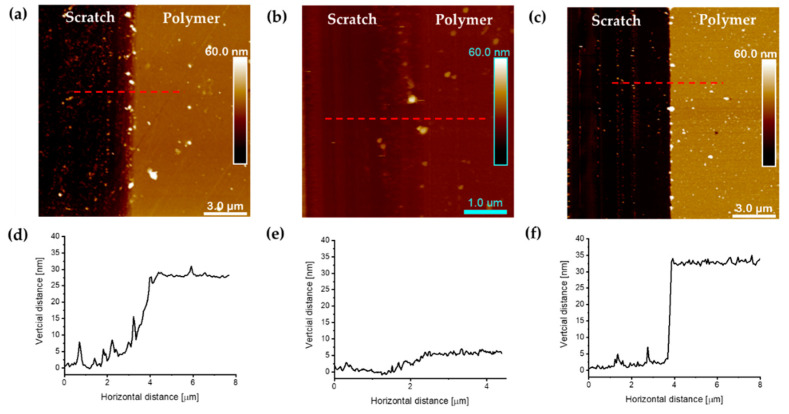
AFM analysis of well-defined polymer brushes grafted from silicon wafers. Height image of PHEA (**a**) according to Table 1, entry 1, (**b**) according to Table 1, entry 2, and (**c**) height image of PHEA-*b*-P*t*BA brushes (according to Table 1, entries 1 and 4). Cross-section profiles (captured in the places marked with a red dotted line in (**a**), (**b**), and (**c**)) for (**d**) PHEA (Table 1, entry 1), (**e**) PHEA (Table 1, entry 2), and (**f**) PHEA-*b*-P*t*BA.

After grafting of polymer brushes from the silicon surface, an analogous reaction without the addition of a sacrificial initiator was investigated (Table 1, entry 2). Interestingly, the AFM measurement showed that the thickness of the obtained polymer layer was only 4 nm (Figure 2e) or 6 nm (Figure S13) and decreased nearly 7-fold in relation to the reaction with the addition of the sacrificial initiator (thickness = 28 nm) (compare Table 2, entry 1 and entry 2). Additionally, WCA slightly decreased from 73.0° ± 1.2 to 68.7° ± 1.4 (Table S5, entry 2 and Figure S12k), in with agreement with the results obtained for the sample with the 28 nm layer of polymer (more hydrophilic, lower WCA), and the literature data for grafted hydrophilic PHEA segments [76].

It seems that the sacrificial initiator-assisted process is characterized by higher efficiency. The addition of the free initiator to the reaction mixture supports the regeneration of the active form of the complex, which makes polymerization more controlled and slower [45,67]. In sacrificial initiator-assisted polymerization, the free initiator in the reaction mixture participates in establishing EC’ (electrolysis-catalysis) balance while the concentration of the surface-attached initiator is not sufficient to effectively contribute to this effect (Scheme 1). Presumably, in the case of synthesis without the presence of the sacrificial initiator, the reduced control over the reaction leads to faster terminations of the growing radicals and lower polymerization efficiency.

### 3.4. AFM Analysis of Polyacrylate Brushes Grafted from Silicon Wafers

In order to determine the thickness of the synthesized polymer layer, AFM measurements were performed. Atomic force microscopy investigation confirmed successful grafting of polymer brushes from silicon wafers and indicated a significant effect of the presence of the sacrificial initiator on the grafting process efficiency. 

Surface imaging allows us to present the direct differences between the scratched surface and the polymer-coated silicon wafer. In Figure 2a–c, the AFM height image of grafted PHEA and PHEA-*b*-P*t*BA brushes is presented. The dark region (left side) of the picture illustrates the scratched area of the sample, whereas the layer of the grafted brushes is shown on the right side. Representative cross section profiles of the studied samples are presented in Figure 2d–f. The lowest point observed in the AFM profiles indicate the uncoated silicon wafer. The measured thicknesses of the grafted layer were 28 ± 1 nm for PHEA synthesized via sacrificial initiator-assisted SI-*se*ATRP and 4 ± 1 nm for the brushes synthesized without the sacrificial initiator. The thickness of the diblock PHEA-*b*-P*t*BA copolymer brushes was found to be equal to 33 ± 1 nm.

### 3.5. Characteristics of Wettability of Modified Silicon Surfaces

In order to examine the wettability properties of the modified surfaces and to compare them with the unmodified brominated silicon wafer, the contact angles on the surfaces of the obtained brushes were determined using liquids of different polarity (water, formamide, diiodomethane) (Table S5, Figure S12). The experimental value of the water contact angle for a silicon wafer covered with hydrophilic PHEA brushes (61.9° ± 1.2 and FSE ~44.8 (± 0.3)–46.4 (±0.1) Table S6, entry 1) was lower than that for wafers with previously attached bromine initiator (73.0° ± 1.2 and FSE ~ 42.6 (± 0.2)–44.0 (±0.1), Table S6, entry 5). On the contrary, the chain extension by hydrophobic P*t*BA segment caused a significant increase in the contact angle value (up to 98.6° ± 1.4 and FSE ~ 36.5 (± 0.2)–37.2 (±0.1), Table S6, entry 4) characteristic of hydrophobic materials [78,79]. The results confirm the correlations between the surface properties of the obtained polymer materials and the chemical nature of the compounds used for modification. Therefore, such surface treatment can be successfully used to obtain hybrid materials, e.g., composed of inorganic supports decorated by organic brushes, with tailored balance between hydrophilic and hydrophobic properties.

## 4. Conclusions

For the first time, poly(2-hydroxyethyl acrylate) was grafted from silicon wafers using sacrificial initiator-assisted ultralow ppm SI-*se*ATRP. The AFM results highlighted the beneficial impact of the sacrificial initiator on the growth of the PHEA brushes. In comparison to the first literature reports concerning HEA polymerization, we applied more than 1000-fold lower copper catalyst loading, which resulted in well-defined PHEA brushes. The preservation of the chain-end functionality of the grafted polymeric chains was confirmed by a chain extension reaction, resulting in a 33 nm brush layer. A significantly reduced amount of the catalyst (from 26 to 6 ppm of Cu) and the application of electric current to the regeneration of the deactivated catalytic forms make the presented technique all-purpose and environmentally friendly. Hence, the application of sacrificial initiator-assisted ultralow ppm SI-*se*ATRP can be a useful tool in the facile synthesis of novel materials precisely decorated with polymer brushes. In particular, in the context of the reduced amount of copper used in the fabrication process, the presented method may be attractive for the synthesis of biocompatible polymers and hybrid materials containing PHEA, poly(*N*, *N*-dimethylaminoethyl methacrylate) (PDMAEMA) or other homopolymer or copolymer brushes. One commercially applied hybrid material consisting of polymer brushes is an organic electrochemical transistor (OECT), in which ions penetrate the polymer coating and affect its conductivity and can convert ionic signals into electronic signals [80]. Therefore, such transistors show high application potential, especially in bionanoelectronics, e.g., biosensors.

## Figures and Tables

**Table 1:** Summary of homopolymer and block copolymer chains generated in solution during sacrificial initiator-assisted ultralow ppm SI-*se*ATRP.

Entry		M	[Cu^II^Br_2_/TPMA] [Ppm By Wt.]	*E*_app_^(a)^	DP_th_^(b)^	*k_p_*^app^^(c)^ [h^−1^]	*k_red_*^app^^(d)^ [s^−1^]	*M*_*n*,app_^(e)^	*M*_*n*,th_^(f)^	*Ð* ^(e)^
**Homopolymer**	1	HEA	26	190 mV (*E*_pc_−120 mV)	460	0.107	0.0009	30,500	72,900	1.31
	2	HEA	26	196 mV (*E*_pc_ −120 mV)	−	−	0.0013	−	−	−
	3 ^(f)^	HEA	26	230 mV (*E*_pc_ −120 mV)	−	−	0.0003	−	−	−
**Second block of copolymer brushes**	4	*t*BA	6	120 mV (*E*_pc_ −80 mV)	388	0.058	0.0005	39,200	49,900	1.81

General reaction conditions: T = 55 °C; *V*_tot_ = 70 mL; entry 1: t = 7.5 h (except entry 2–3: t = 6 h); [HEA]_0_ = 2.4 M; [*t*BA]_0_ = 2.6 M; [EBiB]_0_ = 2.4 mM (except entry 4: [EBiB]_0_ = 2.7 mM); [TBAP]_0_ = 0.2 M. SI-*se*ATRP under constant potential electrolysis conditions (WE = Pt mesh, CE = Al wire (*l* = 10 cm, *d* = 1 mm), RE = Ag/AgI/I^−^); ^(a)^ *E*_app_ was selected based on CV analysis of the catalyst complex (according to Table 1, entry 1 and Figure S3); ^(b)^ DP_th_ = monomer conversion × DP_target_, where DP_target_ = 1000; ^(c)^ monomer conversion and apparent propagation rate coefficients (*k*_p_^app^) were determined by NMR [70]; ^(d)^ apparent reduction rate coefficient (*k*_red_^app^) was determined from a first order plot of current vs. polymerization time (Figure S7); ^(e)^ *M*_n,th_ = ([M]_0_/[I]_0_) × monomer conversion × *M*_monomer_ + *M*_initiator_; ^(f)^ apparent *M*_n_ and *Đ* were determined by THF GPC with PS standards. ^(f)^ Entry 3 (analogous to entry 2) was carried out to confirm the thickness of grafted brushes.

**Table 2:** Summary of AFM and contact angle analysis of homopolymer and block copolymer brushes grafted from the silicon surface via ultralow ppm SI-*se*ATRP.

Entry (According to Table 1)	M	Thickness ^(a)^ [nm]	Θ [°]	FSE ^(b)^ [mJ/m^2^]
1	HEA	28 ± 1	61.9 ± 1.2	44.8 (±0.3)–46.4 (±0.1)
2	HEA	4 ± 1	68.7 ± 1.4	43.1 (±0.4)–43.7 (±0.1)
3	HEA	6 ± 1	−	-
4	*t*BA	5 ± 1 (33 ± 1)	98.6 ± 1.4	36.5 (±0.2)–37.2 (±0.1)

^(a)^ Thickness was determined by AFM measurements (values refer to the total thickness of the bromosilane functionalized layer together with polymer layers). ^(b)^ The free surface energy (FSE, $\gamma_{s}$) was calculated based on the water contact angle (Table S5) according to the Owens–Wendt and van Oss–Good methods (Table S6) [65].

## Supplementary Materials

The following are available online at https://www.mdpi.com/1996-1944/13/16/3559/s1. Figure S1: Cyclic voltammetry of Cu^II^Br_2_/TPMA (black) and in the presence of EBiB (grey) according to (a) Table S1, entry 1, (b) Table S1, entry 2, and (c) Table S1, entry 3. The arrow (green) indicates the applied potential during electrolysis. Figure S2: Current profile vs. polymerization time for the grafting of PHEA brushes from silica wafers via sacrificial initiator-assisted ultralow ppm SI-*se*ATRP according to (a) Table S1, entry 1, (b) Table S1, entry 2, and (c) Table S1, entry 3. Figure S3: Cyclic voltammetry of Cu^II^Br_2_/TPMA (black) and in the presence of EBiB (green). The arrow (red) indicates the applied potential during preparative electrolysis. Measurement conditions: [HEA]_0_/[EBiB]_0_/[Cu^II^Br_2_]_0_/[TPMA]_0_ = 1000/1/0.05/0.10, [HEA]_0_ = 2.2 M, [Cu^II^Br_2_/TPMA]_0_ = 0.12 mM, [TBAP]_0_ = 0.2 M. Table 1, entry 1. Figure S4: Current profile vs. time for the grafting of polymer brushes from silicon wafers according to (a) Table 1, entry 1, (b) Table 1, entry 2, (c) Table 1, entry 3, and (d) Table 1, entry 4. Figure S5: First-order kinetic plot of monomer conversion vs. polymerization time according to: (a) Table S1, entry 1, (b) Table S1, entry 2, and (c) Table S1, entry 3. Figure S6: First-order plot of current vs. polymerization time for the grafting of PHEA brushes from silicon wafers via sacrificial initiator-assisted ultralow ppm SI-*se*ATRP according to (a) Table S1, entry 1, (b) Table S1, entry 2, and (c) Table S1, entry 3. Figure S7: First-order plot of current vs. polymerization time for the grafting of polymer brushes from silicon wafers according to (a) Table 1, entry 1, (b) Table 1, entry 2, (c) Table 1, entry 3 and (d) Table 1, entry 4. Figure S8: ^1^H NMR spectrum of PHEA homopolymer (Table 1, entry 1). Figure S9: Synthesis of well-defined P*t*BA chains generated in the solution: (a) First-order kinetic plot of monomer conversion vs. polymerization time, (b) *M*_n_ and *M*_w_/*M*_n_ vs. monomer conversion. Table 1, entry 4. Figure S10: ^1^H NMR spectrum of P*t*BA homopolymer (Table 1, entry 4). Figure S11: GPC traces of free polymers generated from sacrificial initiator during preparation of surface-grafted PHEA brushes according to (a) Table 1, entry 1 and (b) Table 1, entry 4., Figure S12: (a) Diiodomethane, (e) formamide and (i) water contact angle images of Si-*g*-PHEA prepared according to Table 1 (entry 1), (b) diiodomethane, (f) formamide and (j) water contact angle images of Si-*g*-PHEA prepared according to Table 1 (entry 2), (c) diiodomethane, (g) formamide and (k) water contact angle images of Si-*g*-(PHEA-*b*-P*t*BA) prepared according to Table 1 (entry 4) and (d) diiodomethane, (h) formamide and (l) water contact angle images of Si-Br (prepared according to section: Surface functionalization of silicon wafers). Figure S13: AFM analysis of well-defined polymer brushes grafted from silicon wafers. Height image of PHEA (a) according to Table 1, entry 3, (b) cross-section profile captured in the place marked with a red dotted line in the topography image, (Table 1, entry 3). Table S1: Low ppm *se*ATRP of HEA. Table S2: Theoretical Al^3+^ concentration in solution and polymer by monomer conversion. Table S3: Calculation of Cu^I^/Cu^II^ ratio for the preparation of polyacrylate brushes. Table S4: Calculation of the Dead Chain Fraction (DCF). Table S5: Experimental values of contact angles for synthesized polymer brushes and brominated silica wafer. Table S6: Parameters of free surface energy (FSE) as calculated by Owens–Wendt and van Oss–Good methods for synthesized polymer brushes and brominated silica wafer.
