# Supplementary material for: Polymer Brushes via Surface-Initiated Electrochemically Mediated ATRP: Role of a Sacrificial Initiator in Polymerization of Acrylates on Silicon Substrates
Source: Materials (Basel). 2020 Aug 12;13(16):3559. doi: 10.3390/ma13163559 (PMC7475859; doi:10.3390/ma13163559)
Supplement: Supplementary file 1 [file materials-13-03559-s001.pdf]

materials

Supporting Information

Polymer Brushes via Surface-Initiated Electrochemically Mediated ATRP: Role of a Sacrificial Initiator in Polymerization of Acrylates on Silicon Substrates

Monika Flejszar [1], Paweł Chmielarz [1,*], Karol Wolski [2], Gabriela Grześ [2] and Szczepan Zapotoczny [2]

[1] Department of Physical Chemistry, Faculty of Chemistry, Rzeszow University of Technology, Al. Powstańców Warszawy 6, 35-959 Rzeszów, Poland; d442@stud.prz.edu.pl

[2] Faculty of Chemistry, Jagiellonian University, Gronostajowa 2, 30-387 Kraków, Poland; wolski@chemia.uj.edu.pl (K.W.); gabriela.grzes@doctoral.uj.edu.pl (G.G.); zapotocz@chemia.uj.edu.pl (S.Z.)

* Correspondence: p_chmiel@prz.edu.pl; Tel.: +48-17-865-1809 (P.C.)

Received: 27 July 2020; Accepted: 10 August 2020; Published: date

Additional Experimental Data

Table S1. Low ppm *se*ATRP of HEA.

Entry	t [h]	NaBr [mol / dm³]	HEA /solvent [v/v]	[Cu^{II}Br$_2$/TPMA] [ppm by wt.]	E_{app} (a)	conv. (b) [%]	DP_{theo}	DP_{app} (c)	k_p^{app} (c) [h^{-1}]	k_{red}^{app} (d) [s^{-1}]	$M_{n,app}$ (e)	$M_{n,th}$ (f)	$Đ$ (e)
1	4.5	-	50/50	51	320 mV (E_{pc}– 80 mV)	27	270	274	0.099	0.0021	32 700	43 500	1.35
2	8	0.1	50/50	51	190 mV (E_{pc}– 80 mV)	22	220	220	0.040 (0.051)c)	0.0016	27 600	35 000	1.63
3	4.5	-	25/75	26	180 mV (E_{pc}– 120 mV)	31	310	308	0.094	0.0018	37 100	48 700	1.86

General reaction conditions: T = 55 °C; V_{tot} = 70 mL; [HEA]$_0$ = 2.4 M; [EBiB]$_0$ = 4.8 mM (except entry 3: [EBiB]$_0$ = 2.4 mM); [TBAP]$_0$ = 0.2 M *se*ATRP under constant potential electrolysis (working electrode (WE) = Pt plate, counter electrode (CE) = Al wire (l = 10 cm, d = 1 mm), reference electrode (RE) = Ag/AgI/I$^-$). (a) Applied potential (E_{app}) was selected based on cyclic voltammetry (CV) analysis of catalyst complex (Figure S1a-c, Supporting Information); (b) Monomer conversion and apparent propagation rate coefficients (k_p^{app}) were determined by NMR; (c) k_p^{app} value determined for 4h of reaction time; (d) k_{red}^{app} – apparent reduction rate coefficient determined from first order plot of current vs. time (Figure S6a–c, Supporting Information); (e) apparent M_n and $Đ$ were determined by THF GPC with PS standards; (f) $M_{n,th}$ = ([M]$_0$/[I]$_0$) × monomer conversion × $M_{monomer}$ + $M_{initiator}$.

Table S2. Theoretical Al^{3+} concentration in solution and polymer by monomer conversion.

Entry		Entry	Q (a) (C)	$n_{Al^{3+}}$ (b) (mol × 10^5)	[Al^{3+}]$_{solution}$ (c) (ppm by wt.)	[Al^{3+}]$_{polymer}$ (d) (ppm by wt.)
according to Table 1	1	1	3.579	1.24	4.6	0.5
	2	2	6.585	2.27	8.5	0.8
	4	3	1.699	0.59	2.3	0.4
according to Table S1	1	4	0.579	0.20	3.2	0.4
	2	5	2.989	1.03	16.6	1.8
	3	6	1.835	0.63	10.4	0.8

(a) The total passed charge was calculated by integration of the chronoamperometry (CA) area, that is, $Q = I \times t$; (b) Theoretical amount of Al^{3+} in the reaction mixture was calculated from CA: $n_{Al^{3+}} = Q/F/3$, where F = 96,485 C/mol; (c) The Al concentrations in the reaction mixture was calculated according to the equation defined as: [Al^{3+}]$_{solution}$ = [Al^{3+}] × MW_{Al} / wt_{total} × 1,000,000; (d) The Al concentrations in pure polymer sample were determined by the monomer conversion, i.e., [Al^{3+}]$_{polymer}$ = [Al^{3+}]$_{solution}$/df × monomer conversion, where df is dilute factor df = 4 (except entries 3: df = 2.5; for entries 4–5: df = 2).

Table S3. Calculation of Cu^I/Cu^{II} ratio for the preparation of polyacrylate brushes.

Entry		Entry	k_p^{app} (a) (h^{-1})	[P_n•] (a) (M × 10^9)	K_{ATRP} (b) (×10^7)	[P_n-Br] (M)	[Cu^I]/[Cu^{II}] (c)	[$Cu^I TPMA^+$] (%)	[Br-$Cu^{II}TPMA^+$] (%)
according	1	1	0.107	8.49	1.30	0.0024	27	96.5	3.52
to Table 1	4	2	0.058	4.60	1.30	0.0028	13	92.8	7.22
according	1	3	0.099	7.86	1.30	0.0048	13	92.7	7.30
to Table	2	4	0.040	3.17	1.30	0.0048	5	83.7	16.32
S1	3	5	0.094	7.46	1.30	0.0024	24	96.0	3.98

(a) The radical concentration $[P_n^\bullet]$ was calculated according to the equation defined as $[P_n^\bullet] = \left(\frac{d\ln[M]}{dt}\right)(k_p)^{-1}$ [1], where $\frac{d\ln[M]}{dt}$ values were calculated from the first order kinetics plots (Figures S7, 1a and 2a) [2], $k_p = 3{,}500\ M^{-1}s^{-1}$ [3]. (b) $K_{ATRP} = 1.3 \times 10^{-8}$ was determined for the $Cu^I/TPMA^+$ catalyst in methyl acrylate/acetonitrile 50/50 (v/v) at 50 °C [4]. (c) The Cu^I/Cu^{II} ratio was calculated according to the equation defined as $\frac{[Cu^I TPMA^+]}{[Br{-}Cu^{II}TPMA^+]} = \frac{[P_n^\bullet]}{[P_n{-}Br]K_{ATRP}}$ [4].

Table S4. Calculation of Dead Chain Fraction (DCF).

Entry		Entry	[P_n•] (a) (M × 10^9)	[D] (b) (M × 10^4)	[P_n-Br]$_0$ (M)	DCF (c) (%)	CEF (d) (%)
according to Table 1	1	1	8.49	1.9	0.0024	7.9	92.1
	4	2	4.60	3.3	0.0028	11.8	88.2
	1	3	7.86	1.2	0.0048	2.5	97.5
according to Table S1	2	4	3.17	0.3	0.0048	0.7	99.3
	3	5	7.46	1.1	0.0024	4.5	95.5

(a) The radical concentration $[P_n^\bullet]$ was calculated according to the equation defined as $[P_n^\bullet] = \left(\frac{d\ln[M]}{dt}\right)(k_p)^{-1}$ [1], where $\frac{d\ln[M]}{dt}$ values were calculated from the first order kinetics plots (Figure S7, 1a and 2a) [2], $k_p = 3.5 \cdot 10^3\ M^{-1}s^{-1}$ [3]. (b) The concentration of terminated chains [D] was calculated according to the equation defined as $[D] = k_t[P\cdot]^2 t$ where t (denote reaction time) = s, $k_t = 1.2 \times 10^8\ M^{-1}s^{-1}$ was determined for the 2-hydroxethyl acrylate at room temperature [5] (except entry 2: $k_t = 5.7 \times 10^8\ M^{-1}s^{-1}$ was determined for the *n*-butyl acrylate at 50 °C) [6]. (c) DCF = $\left(\frac{[D]}{[P{-}Br]_0}\right) \cdot 100\%$ [1]. (d) CEF = 100% − DCF.

Table S5. Experimental values of contact angles for synthesized polymer brushes and brominated silica wafer.

Entry (according to Table 1)	Experimental Values of Θ [°]					
	Water	Standard Deviation	Formamide	Standard Deviation	Diiodomethane	Standard Deviation
1	61.86	1.23	45.23	1.06	39.26	1.32
2	68.67	1.40	50.28	1.63	38.68	1.99
4	98.60	1.43	69.60	1.94	46.15	1.06
5 (Si-Br)	73.01	1.19	54.68	1.27	34.22	1.12

Table S6. Parameters of free surface energy (FSE) as calculated by Owens-Wendt and van Oss-Good methods for synthesized polymer brushes and brominated silica wafer.

Entry. (according to Table 1)	Parameters of FSE [mJ/m²]							
	Owens-Wendt Method			van Oss-Good Method				
	Water-diiodomethane			Diiodomethane-formamide-water				
	γ_s	γ_s^d	γ_s^p	γ_s^{LW}	γ_{s^+}	γ_{s^-}	γ_s^{AB}	γ_s
1	46.36 (±0.09)	31.82 (±0.08)	14.54 (±0.05)	39.986 (±0.9)	0.329 (±0.003)	17.534 (±0.249)	4.806 (±0.044)	44.791 (±0.263)
2	43.68 (±0.13)	33.73 (±0.12)	9.95 (±0.04)	40.268 (±0.393)	0.161 (±0.002)	12.685 (±0.222)	2.858 (±0.034)	43.126 (±0.395)
4	37.23 (±0.08)	37.08 (±0.08)	0.152 (±0.001)	36.392 (±0.189)	0.0122 (±0.0001)	0.243 (±0.004)	0.109 (±0.001)	36.501 (±0.189)
5 (Si-Br)	44.03 (±0.08)	37.07 (±0.08)	6.96 (±0.03)	42.386 (±0.233)	0.00077 (±0.00001)	10.674 (±0.152)	0.182 (±0.001)	42.568 (±0.233)

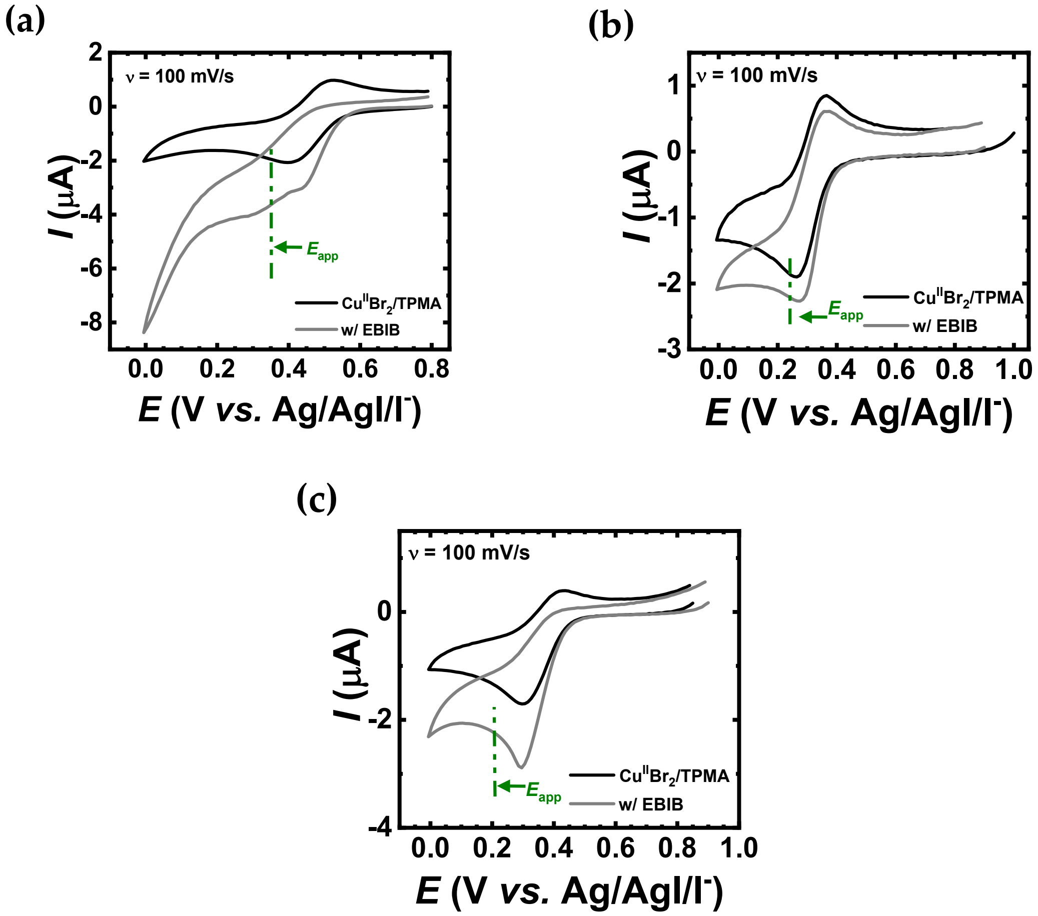

Figure S1. Cyclic voltammetry of $Cu^{II}Br_2$/TPMA (black) and in the presence of EBiB (grey) according to (**a**) Table S1, entry 1, (**b**) Table S1, entry 2, and (**c**) Table S1, entry 3. The arrow (green) indicates the applied potential during electrolysis.

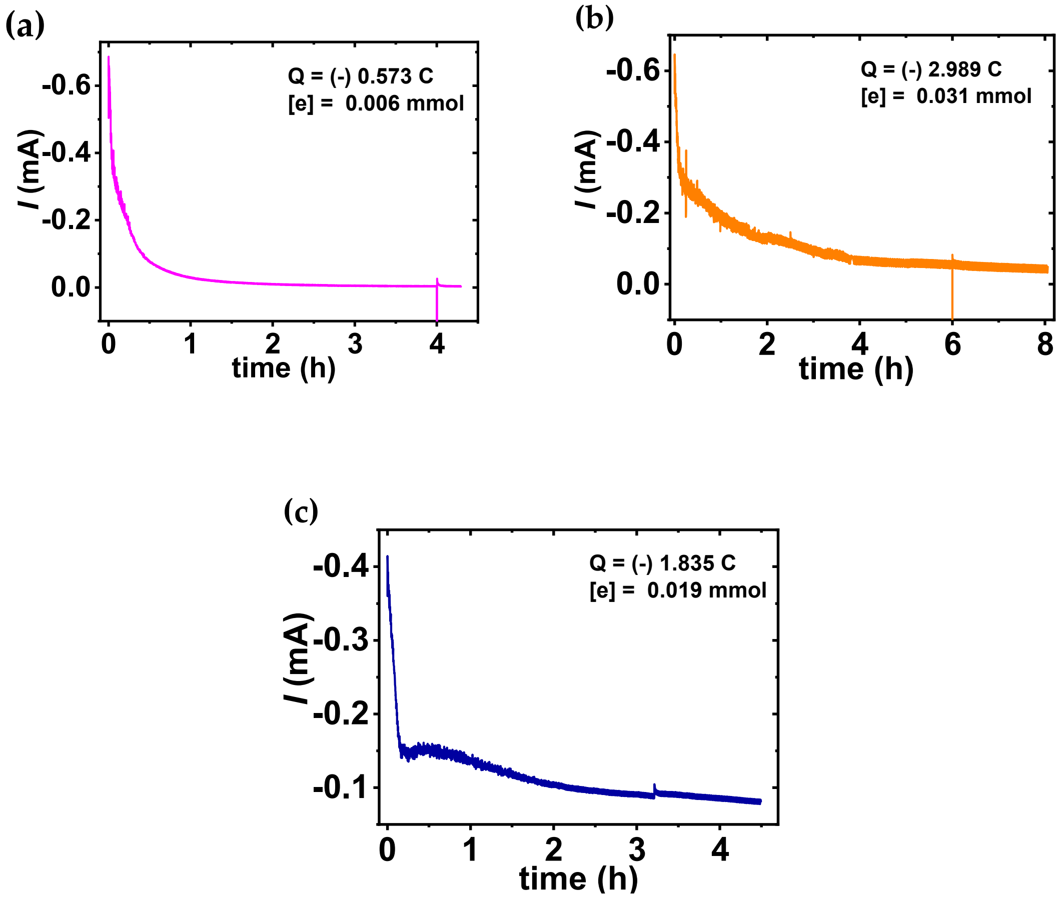

Figure S2. Current profile vs. polymerization time for the grafting of PHEA brushes from silica wafers *via* sacrificial initiator-assisted ultralow ppm SI-*se*ATRP according to (**a**) Table S1, entry 1, (**b**) Table S1, entry 2, and (**c**) Table S1, entry 3.

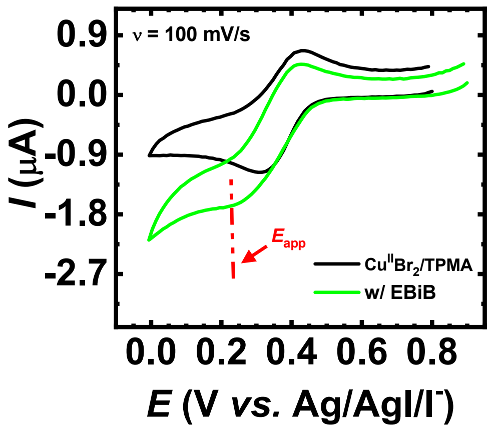

Figure S3. Cyclic voltammetry of $Cu^{II}Br_2$/TPMA (black) and in the presence of EBiB (green). The arrow (red) indicates the applied potential during preparative electrolysis. Measurement conditions: $[HEA]_0/[EBiB]_0/[Cu^{II}Br_2]_0/[TPMA]_0$ = 1000/1/0.05/0.10, $[HEA]_0$ = 2.2 M, $[Cu^{II}Br_2/TPMA]_0$ = 0.12 mM, $[TBAP]_0$ = 0.2 M. Table 1, entry 1.

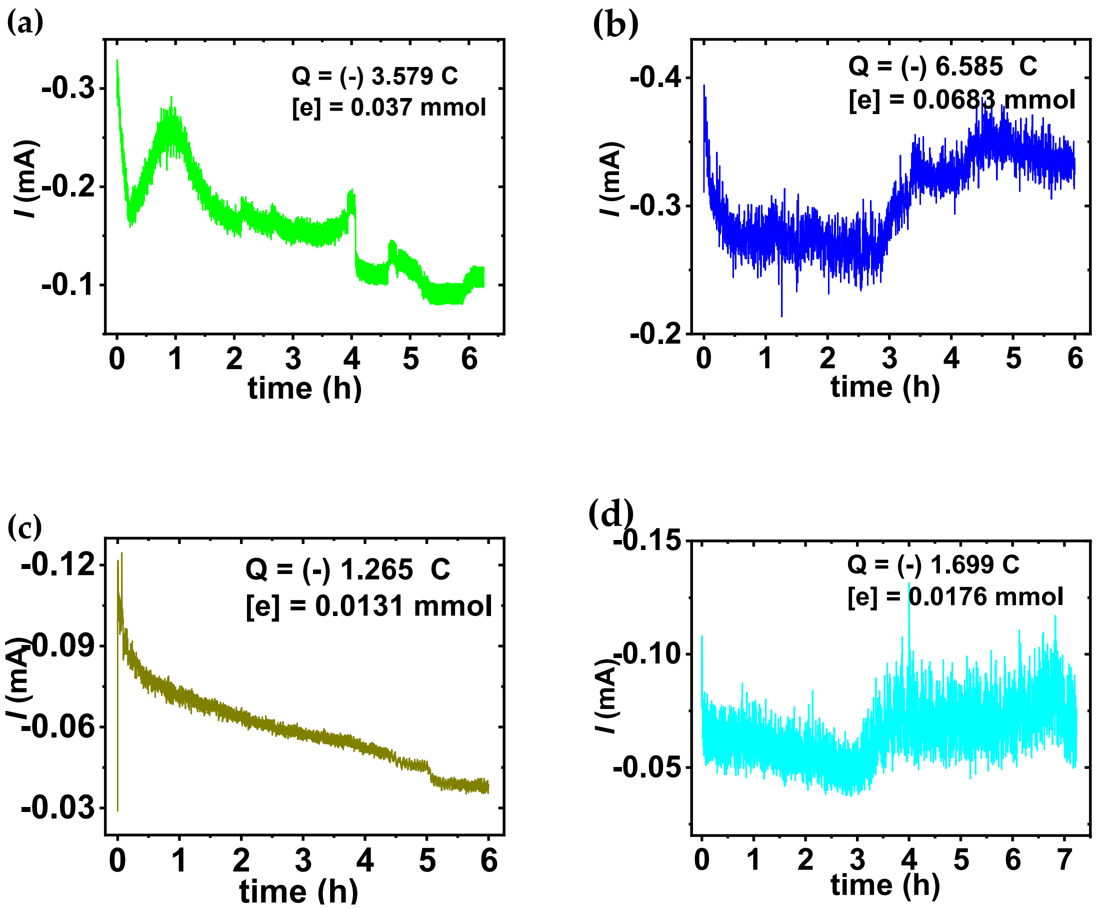

Figure S4. Current profile vs. time for the grafting of polymer brushes from silicon wafers according to (**a**) Table 1, entry 1, (**b**) Table 1, entry 2, (**c**) Table 1, entry 3, and (**d**) Table 1, entry 4.

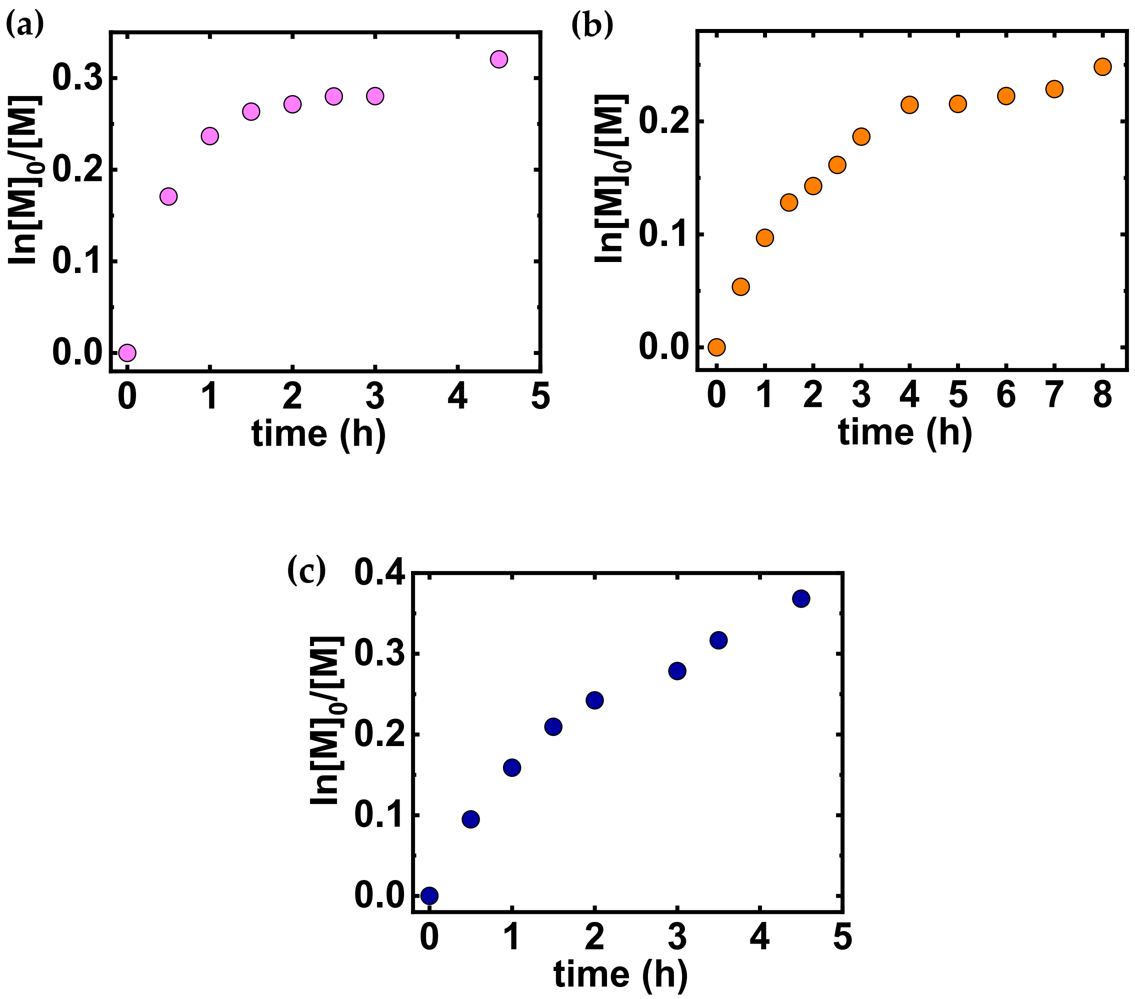

Figure S5. First-order kinetic plot of monomer conversion vs. polymerization time according to: (**a**) Table S1, entry 1, (**b**) Table S1, entry 2, and (**c**) Table S1, entry 3.

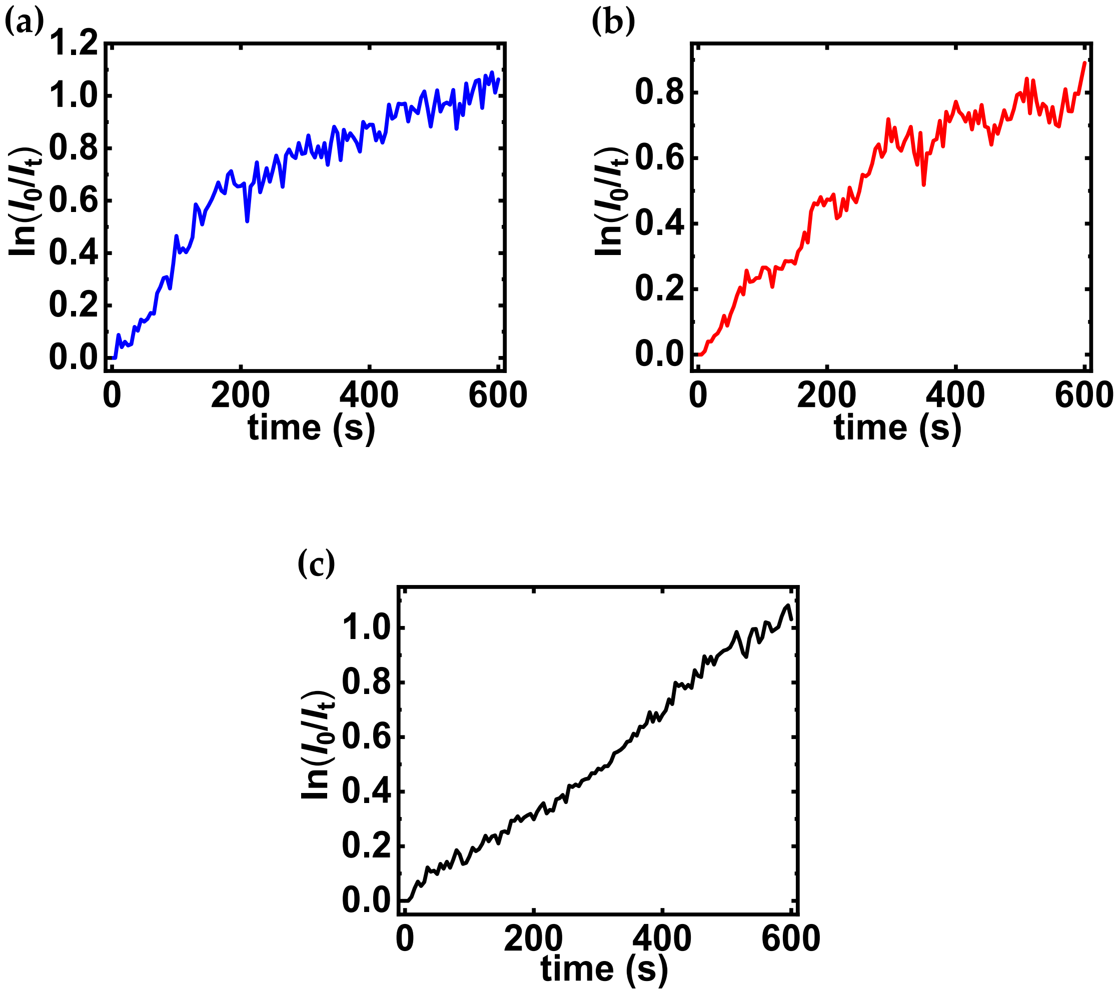

Figure S6. First-order plot of current vs. polymerization time for the grafting of PHEA brushes from silicon wafers via sacrificial initiator-assisted ultralow ppm SI-*se*ATRP according to (**a**) Table S1, entry 1, (**b**) Table S1, entry 2, and (**c**) Table S1, entry 3.

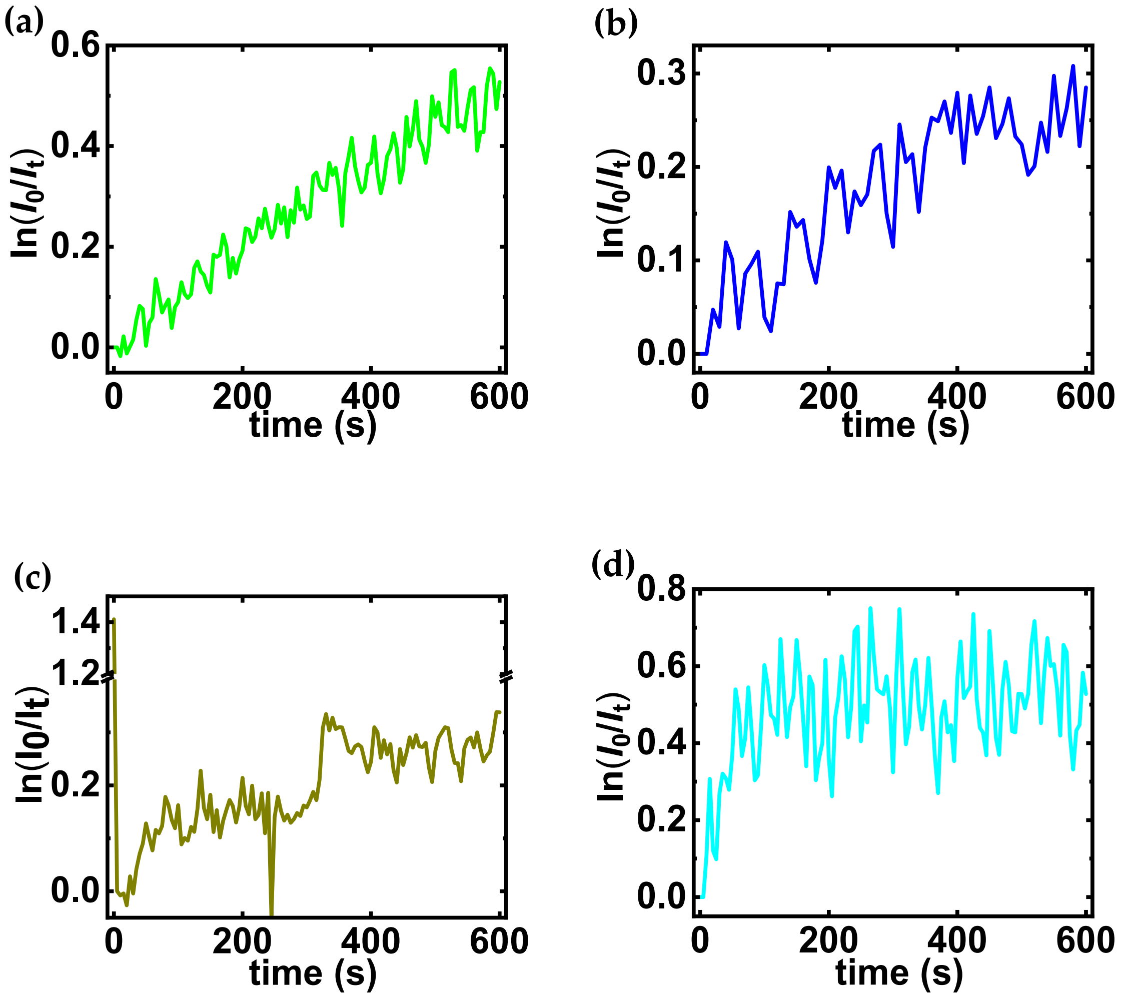

Figure S7. First-order plot of current *vs.* polymerization time for the grafting of polymer brushes from silicon wafers according to (**a**) Table 1, entry 1, (**b**) Table 1, entry 2, (**c**) Table 1, entry 3 and (**d**) Table 1, entry 4.

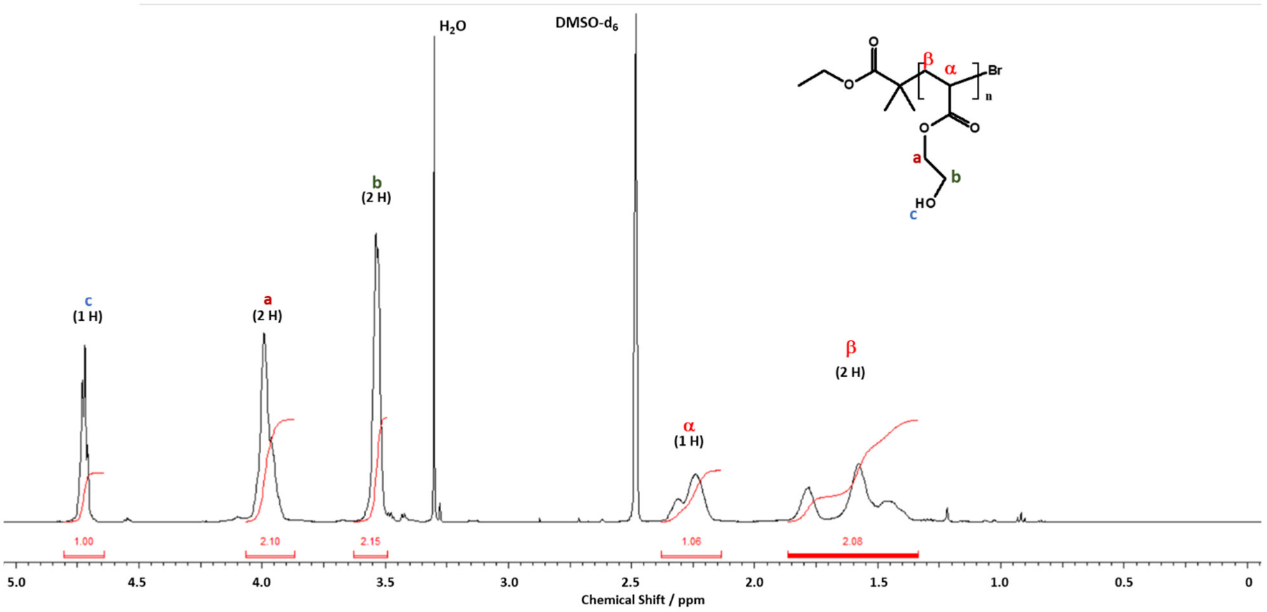

Figure S8. NMR spectrum of PHEA homopolymer (Table 1, entry 1).

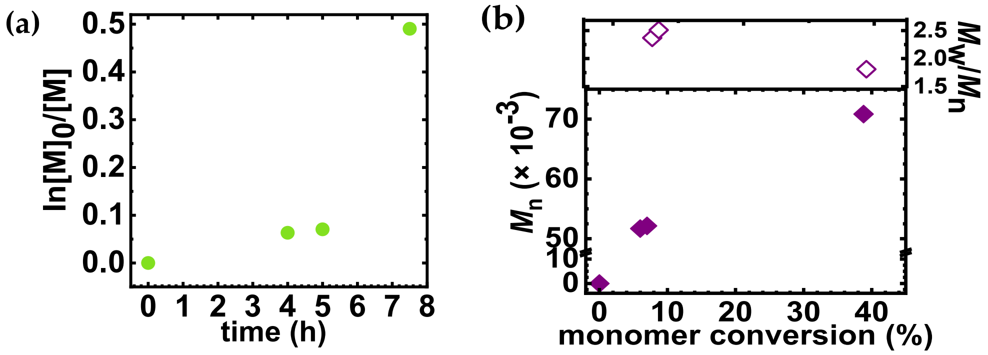

Figure S9. Synthesis of well-defined P*t*BA chains generated in the solution: (**a**) First-order kinetic plot of monomer conversion *vs.* polymerization time, (**b**) M_n and M_w/M_n vs. monomer conversion. Table 1, entry 4.

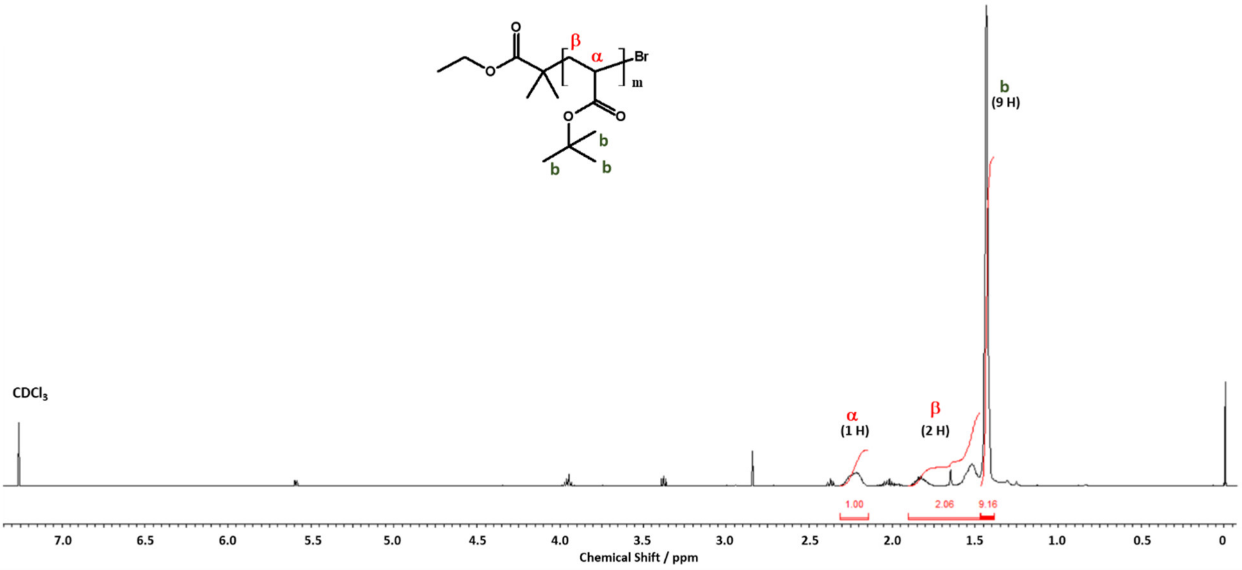

Figure S10. ^{1}H NMR spectrum of P*t*BA homopolymer (Table 1, entry 4).

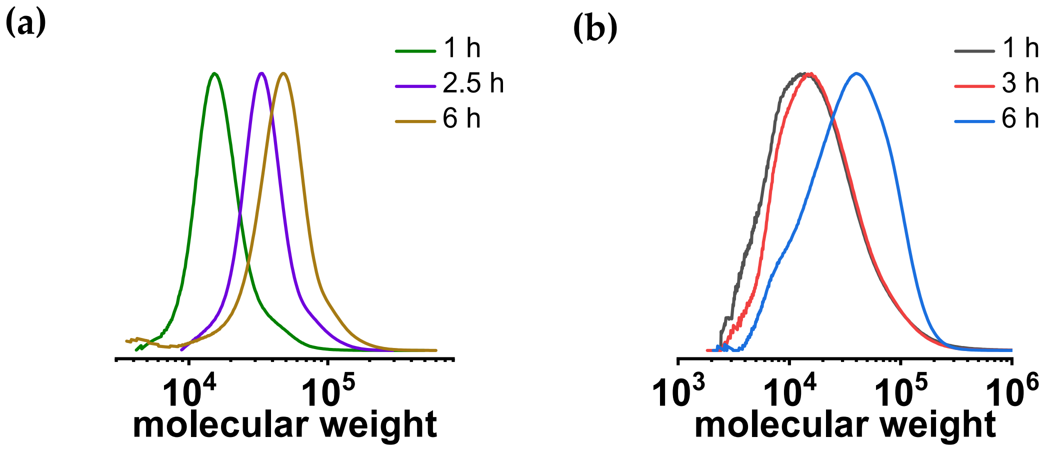

Figure S11. GPC traces of free polymers generated from sacrificial initiator during preparation of surface-grafted PHEA brushes according to (**a**) Table 1, entry 1 and (**b**) Table 1, entry 4.

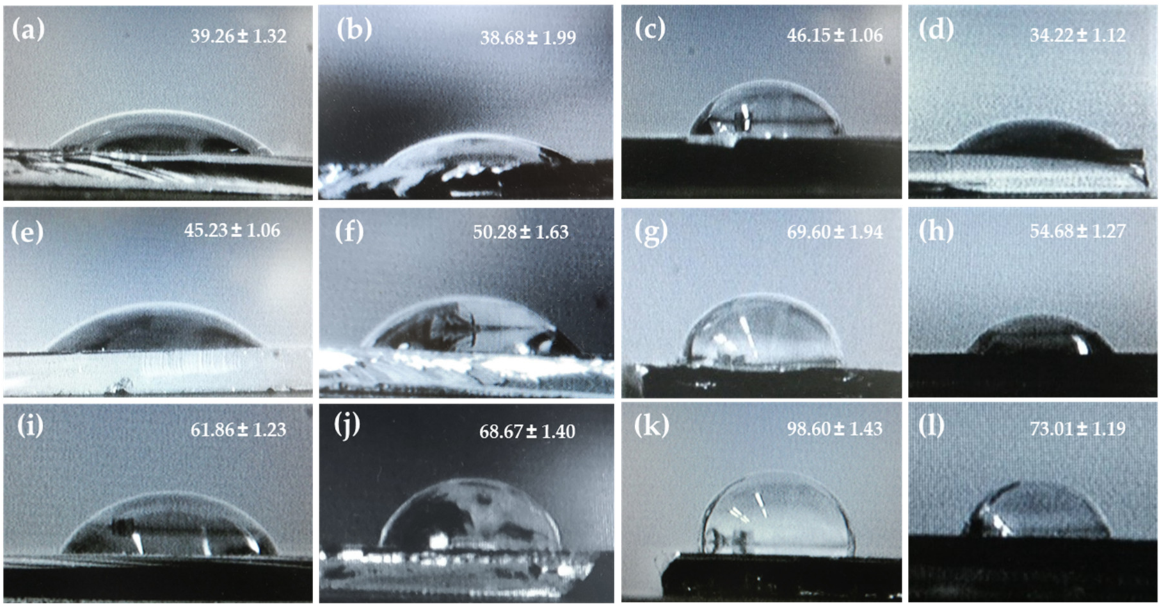

Figure S12. (**a**) Diiodomethane, (**e**) formamide and (**i**) water contact angle images of Si-*g*-PHEA prepared according to Table 1 (entry 1), (**b**) diiodomethane, (**f**) formamide and (**j**) water contact angle images of Si-*g*-PHEA prepared according to Table 1 (entry 2), (**c**) diiodomethane, (**g**) formamide and (**k**) water contact angle images of Si-*g*-(PHEA-*b*-P*t*BA) prepared according to Table 1 (entry 4) and (**d**) diiodomethane, (**h**) formamide and (l) water contact angle images of Si-Br.

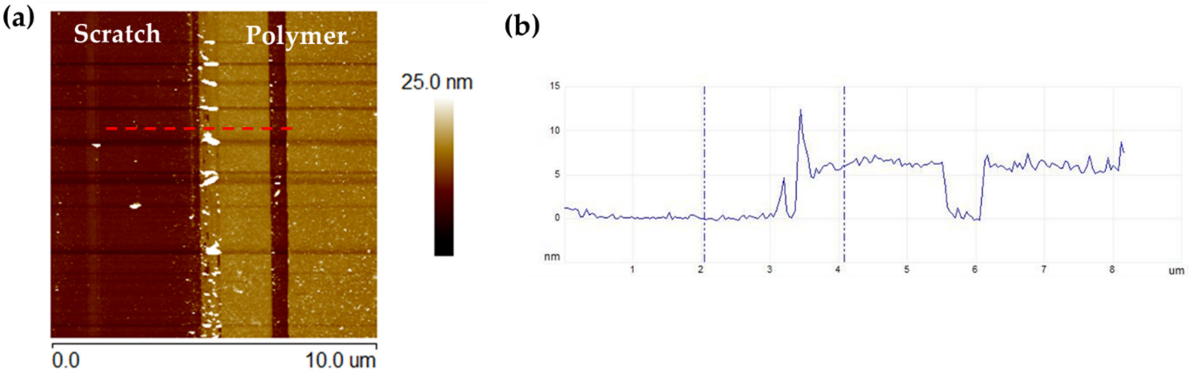

Figure S13. AFM analysis of well-defined polymer brushes grafted from silicon wafers. Height image of PHEA (**a**) according to Table 1, entry 3, (**b**) cross-section profile captured in the place marked with a red dotted line in the topography image, (Table 1, entry 3).

References

1. Zhong, M. J.; Matyjaszewski, K., How fast can a CRP be conducted with preserved chain end functionality? *Macromolecules* **2011,** *44*, 2668–2677.
2. Chmielarz, P.; Krys, P.; Wang, Z. Y.; Wang, Y.; Matyjaszewski, K., Synthesis of well-defined polymer brushes from silicon wafers *via* surface-initiated *se*ATRP. *Macromol. Chem. Phys.* **2017,** *218*, 1700106.
3. Mavroudakis, E.; Liang, K.; Moscatelli, D.; Hutchinson, R. A., A combined computational and experimental study on the free-radical copolymerization of styrene and hydroxyethyl acrylate. *Macromol. Chem. Phys.* **2012,** *213*, 1706–1716.
4. Wang, Y.; Kwak, Y.; Buback, J.; Buback, M.; Matyjaszewski, K., Determination of ATRP equilibrium constants under polymerization conditions. *ACS Macro Letters* **2012,** *1*, 1367–1370.
5. Wojnarovits, L.; Takacs, E., Rate coefficients of the initial steps of radiation induced oligomerization of acrylates in dilute aqueous solution. *Radiat. Phys. Chem.* **1999,** *55*, 639–644.
6. Barth, J.; Buback, M.; Hesse, P.; Sergeeva, T., Termination and transfer kinetics of butyl acrylate radical polymerization studied *via* SP-PLP-EPR. *Macromolecules* **2010,** *43*, 4023–4031.

© 2020 by the authors. Submitted for possible open access publication under the terms and conditions of the Creative Commons Attribution (CC BY) license (http://creativecommons.org/licenses/by/4.0/).
